# Serum biomarker panel for the diagnosis of rheumatoid arthritis

**DOI:** 10.1186/s13075-020-02405-7

**Published:** 2021-01-18

**Authors:** Sora Mun, Jiyeong Lee, Mira Park, Jieun Shin, Mi-Kyoung Lim, Hee-Gyoo Kang

**Affiliations:** 1 Department of Biomedical Laboratory Science, College of Health Sciences, Eulji University, Seongnam, Republic of Korea; 2 Department of Biomedical Laboratory Science, College of Health Sciences, Eulji University, Daejeon, Republic of Korea; 3 Department of Preventive Medicine, School of Medicine, Eulji University, Daejeon, Republic of Korea; 4 Liberal Arts, Woosuk University, Jeonju, Republic of Korea; 5 Division of Rheumatology, Department of Medicine, Eulji University School of Medicine, Daejeon, Republic of Korea

**Keywords:** Rheumatoid arthritis, Angiotensinogen, Biomarkers, Serum amyloid A-4 protein, Retinol-binding protein-4, Vitamin D-binding protein

## Abstract

**Background:**

Rheumatoid arthritis (RA) is an autoimmune disease of inflammatory joint damage, wherein C-reactive protein and autoantibodies including rheumatoid factor (RF) and anti-cyclic citrullinated peptide (anti-CCP) are rapidly elevated. These serological factors are diagnostic markers of RA; however, their sensitivity and specificity for prediction warrant improvement for an early and accurate diagnosis.

**Methods:**

We aimed to identify alternative biomarkers by serum protein profiling using LC-MS/MS. We performed statistical and functional analysis of differentially expressed proteins to identify biomarker candidates complementing conventional serological tests.

**Results:**

Seven biomarker candidates were verified through multiple reaction monitoring-based quantitative analysis, of which angiotensinogen (AGT), serum amyloid A-4 protein (SAA4), vitamin D-binding protein (VDBP), and retinol-binding protein-4 (RBP4) had an area under the curve over 0.8, thus distinguishing RA patients, including seronegative (RF- and anti-CCP-negative) RA patients, from healthy controls.

**Conclusions:**

Therefore, among seronegative RA patients, a four-biomarker panel (AGT, SAA4, VDBP, and RBP4) can prevent false negatives and help diagnose RA accurately.

## Background

Rheumatoid arthritis (RA) is an autoimmune disease [1] with an unknown etiology. However, genetic factors account for 60% of the RA risk factors. Initially, these genetic factors include gene polymorphisms, epigenetic factors including DNA methylation and histone acetylation, and complex factors [2–4]. Other risk factors include environmental factors such as smoking, oral health, and diet [5]. The disease is initially characterized by an inflammatory response, followed by autoantibody activation and damage to the synovial membrane and joints. Activation of inflammation increases cytokine, chemokine, and inflammatory reactants such as C-reactive protein (CRP) [6]. Furthermore, a series of immune responses are triggered with an increase in inflammation. Hence, autoantibodies are overproduced, leading to an increase in immunoglobulin M (RF) and anti-CCP in RA patients [7, 8]. When serum peptides are citrullinated or subjected to other posttranslational modifications by various environmental stimuli, the altered peptides are presented to immune cells, including T cells as antigens, and antibodies such as anti-CCP are produced [5, 7–10]. Therefore, CRP, RF, and anti-CCP, representing the inflammatory and immune response of RA, are diagnostic blood biomarkers [11, 12].

However, existing biomarkers have limitations concerning RA diagnosis. For example, the sensitivity and specificity of RF are 60–90% and 85%, respectively. To improve the efficiency of RA diagnosis, anti-CCP is used with RF. Anti-CCP has higher ACCP positivity than RF positivity among RA patients; however, the sensitivity and specificity of the two markers do not significantly differ [13]. Therefore, novel diagnostic biomarkers complementing the existing biomarkers, i.e., RF and anti-CCP, are required. If these new biomarkers could diagnose seronegative (RF- and anti-CCP-negative) RA patients, they could contribute to the accurate diagnosis and treatment of RA [14, 15]. Furthermore, current diagnostic biomarkers reflect the status of inflammation and immunity among RA patients. New protein biomarkers potentially detected through serum protein profiling are expected to represent various physiological changes in RA, other than inflammation and immunity.

In most previous proteomics studies, blood samples were pooled for MS analysis [16]. However, it is important to analyze individual serum samples to reflect individual alterations in serum protein levels [12, 17], but it is difficult to analyze individual serum samples. First, it is difficult to obtain an adequate volume of individual serum samples. Second, individual MS analysis is costly and time-consuming. Third, clinical data interpretation is challenging owing to the complexity of the status of RA patients. Finally, it is difficult to control the data processes. Nevertheless, when some patients have a high abundance of certain proteins, these protein expression patterns seem to represent all patients. However, this discrepancy can be eliminated through individual sample analysis. Furthermore, MS analysis of individual serum samples is important for biomarker discovery because it helps validate the pattern of differential protein expression among all RA patients. Moreover, serum samples can be classified and validated under various clinical parameters [18].

In a recent study, individual samples were classified under clinical parameters to identify diagnostic biomarkers among seronegative (RF- and anti-CCP-negative) RA patients. In addition, individual sample analysis facilitates the separate analysis of the mild-to-moderate and advanced severe cases. As it is important to assess the clinical course of RA patients, analyses based on the disease status of patients are possible through the analysis of individual samples, thus facilitating the classification of patients under the clinical differences and the concomitant identification of novel biomarkers for accurate RA diagnosis and treatment.

Therefore, in this study, we attempted to develop a biomarker panel to distinguish seronegative (RF- and anti-CCP-negative) RA patients by analyzing the serum proteins relative to those of healthy controls. The experimental cohort was further divided into a discovery and validation cohort. Diagnostic biomarkers were selected from among 50 RA patients and 43 healthy controls in the discovery cohort and from among 251 healthy controls and 230 RA patients in the validation cohort (Table 1).

**Table 1:** Clinical characteristics of patients with rheumatoid arthritis

Characteristic	Healthy controls	Patients with RA
Discovery set		
*n*	43	50
Sex (male/female)	18/25	11/39
Age	56.74 ± 4.34	65.54 ± 8.91
DAS 28	–	2.61 ± 1.12
RF	–	76.96 ± 72.09
Anti-CCP	–	102.22 ± 109.81
Validation set		
*n*	251	230
Sex (male/female)	145/106	33/197
Age	53.87 ± 6.75	61.74 ± 10.80
DAS 28	–	2.58 ± 1.10
RF	–	90.36 ± 158.77
Anti-CCP	–	102.11 ± 108.04

## Methods

### Experimental design and statistical rationale

Serum samples of 251 RA patients and 230 healthy controls for biomarker identification were collected from the Eulji University Hospital Institutional Review Board (EMC 2016-03-019, 31 March 2016). Written informed consent was obtained from all subjects. Participants who were diagnosed by rheumatologists fulfilling the ACR were recruited as the patient group, with no restrictions on gender and age. Healthy controls with previous or current disease history (rheumatoid arthritis, myocardial infarction (MI), angina, stroke, high blood pressure, depression, and/or diabetes mellitus) were excluded for recruitment. Blood was collected in an anticoagulant-free vacutainer. After 2 h at 24 °C, blood samples were centrifuged at 4000×*g* for 5 min to separate the serum. Highly abundant serum proteins including albumin, IgG, antitrypsin, IgA, transferrin, and haptoglobin were depleted using a multiple affinity removal system comprising an LC column (human 6-HC, 4.6 × 50 mm; Agilent Technologies, Santa Clara, CA, USA), as described [12]. The eluted sample containing low-abundance proteins was concentrated using a Nanosep device with a modified polyethersulfone membrane 3 K (Pall, Zaventem, Belgium) and analyzed using a mass spectrometer(AB Sciex 5600, Framingham, MA, USA) to select significant candidate biomarkers. Candidate biomarkers were validated using multiple reaction monitoring (MRM)-based targeted protein quantification.

### Statistical analysis

To select candidate biomarkers, a corrected *p* value, obtained from the Benjamini-Hochberg test, was used, and differentially expressed proteins with a *p* value < 0.05 were used for further analysis. We performed unpaired *t* tests with Welch’s correction using the GraphPad Prism version 8.0 for Windows (GraphPad Software Inc., San Diego, CA) to assess the results of the MRM-based quantification analysis between healthy controls and RA patients; differences with a *p* value < 0.001 were significant. For predicting the classification accuracy of biomarkers, logistic regression analysis was performed using the SPSS software package version 18.0.0 (SPSS Inc., Chicago, IL, USA).

### Determination of protein concentration and tryptic digestion

To determine the serum protein levels for MS analysis, a Bradford assay (Bio-Rad, Hercules, CA, USA) was performed according to the manufacturer’s instructions. Samples containing 100 μg serum proteins were reduced via treatment with 5 mM Tris (2-carboxyethyl) phosphine (Pierce Chemical Company, Rockford, IL, USA) at 37 °C, 300 rpm, for 30 min, followed by treatment with 15 mM iodoacetamide (Sigma-Aldrich, St. Louis, MO, USA) for alkylation at 24 °C, 300 rpm, for 1 h in the dark. Serum proteins were cleaved into peptides, using mass spectrometry-grade trypsin gold (Promega Corporation, Fitchburg, WI, USA) at 37 °C overnight. The cleavage products were cleaned using a C18 cartridge (Waters Corporation, Milford, MA, USA).

### OFFGEL fractionation and LC-MS/MS analysis

The serum proteins in each sample were separated into 12 fractions through pH 3–10 isoelectric points, using the OFFGEL fractionator (3100 OFFGEL Low Res Kit, pH 3–10; Agilent Technologies, Santa Clara, CA, USA) according to the manufacturer’s instructions. Twelve fractions were loaded onto an Eksigent nanoLC 400 system and the cHiPLC® (AB Sciex, Concord, ON, Canada) and analyzed, and the proteins were identified using a TripleTOF 5600 mass spectrometer (AB Sciex). Thereafter, for relative analysis, SWATH acquisition was conducted. In each run, 100 μg/mL of samples was injected onto an Eksigent ChromXP nanoLC trap column (350 μm i.d. × 0.5 mm, ChromXP C18 3 μm) at a flow rate of 5000 nL/min. Samples were eluted from the Eksigent ChromXP nanoLC column (75 μm i.d. × 15 cm) at a flow rate of 300 nL/min for 120 min, and mobile phase B buffer was added gradually into the column (5–90%) over a 120-min total run time. The gradient of mobile phase B buffer was (time and % B) 0 min/mobile phase B 5%, 10.5 min/40%, 105.5 min/90%, 111.5 min/90%, 112 min/5%, and 120 min/5%. Mobile phase B and A buffer, and the search parameters are as described [12].

### Synthesis and purification of label-free standard peptides

Seven candidate proteins were determined as putative diagnostic biomarkers. Peptides for absolute quantification through MRM analysis were selected and synthesized using Peptron (Daejeon, South Korea). These criteria were set for peptide selection: (1) peptides without miscleaved sites, (2) unmodified peptides, (3) peptides not comprising Met, (4) peptides with 7–15 residues, and (6) peptides with a low false discovery rate (FDR) (usually zero). After prototypic tryptic peptide standards were synthesized, two-fold serial dilutions were conducted using 1 mM/μL stock peptide standards in 0.1% formic acid or DMSO, following the manufacturer’s protocol.

### Label-free quantification through MRM analysis

Skyline was used to determine MRM Q1/Q3 ion pairs from selected peptides, as described (Mun et al. [12]). Voltage parameters including collision energy (CE), declustering potential (DP), and cell exit potential (CXP) were determined through compound optimization for each transition. AB Sciex Exion LC was used to segregate the samples using ACQUITY UPLC BEH C18 Column (130 Å, 1.7 μm, 2.1 mm × 150 mm) with an ACQUITY UPLC BEH C18 VanGuard Pre-column (130 Å, 1.7 μm, 2.1 mm × 5 mm). Samples of healthy controls and RA patients were analyzed using AB Sciex QTRAP5500. Each sample was loaded onto the LC column with a gradient of 5–90% mobile phase B for a total run time of 30 min. The mobile phase B buffer was gradually introduced in the LC column: (time/% B) 1 min/mobile phase B 5%, 50 min/40%, 21–25 min/90%, and 25.5–30 min/5%. Mobile phase B comprised 0.1% formic acid in HPLC-grade acetonitrile, and mobile phase A comprised 0.1% formic acid in HPLC-grade water. The source parameters for MRM analysis were curtain gas, 206.84 kPa; low collision gas; ion spray voltage, 5500 V; temperature, 400 °C; ion source gas 1, 275.79 kPa; ion source gas 2, 413.69 kPa.

## Results

**Fig. 1:**
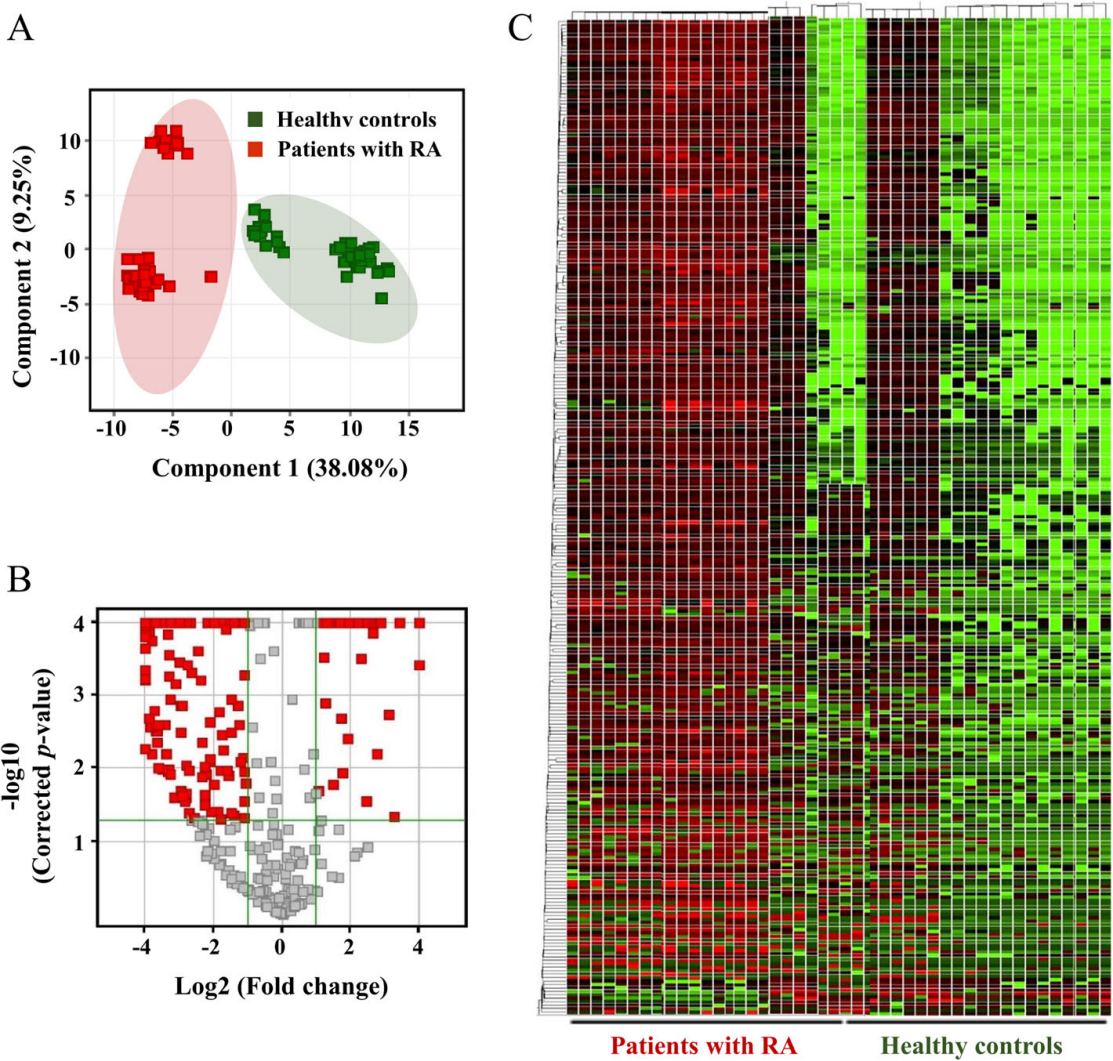
Protein quantification through SWATH acquisition and principal component analysis for group clustering. **a** Venn diagram of the identified proteins among healthy controls and rheumatoid arthritis (RA) patients. **b** Clustering analysis of > 2-fold differentially expressed proteins filtered by the *p* value (*p* < 0.05) on partial least squares discriminant analysis. Differentially expressed proteins by > 2-fold filtered by the *p* value (*p* < 0.05) through the line plot and volcano plot analysis. **c** Heatmap analysis for healthy controls and RA patients

Through a qualitative analysis, performed using SCIEX 5600QTOF, 194 and 111 proteins were uniquely identified from among healthy controls and RA patients, respectively, and 339 proteins were identified in both healthy controls and RA patients. Proteins identified through the IDA method between the two groups were quantified using SWATH acquisition. Principal component analysis (PCA) was performed using the quantification data of each sample. Consequently, healthy controls were distinguished from RA patients (Fig. 1a).

**Fig. 2:**
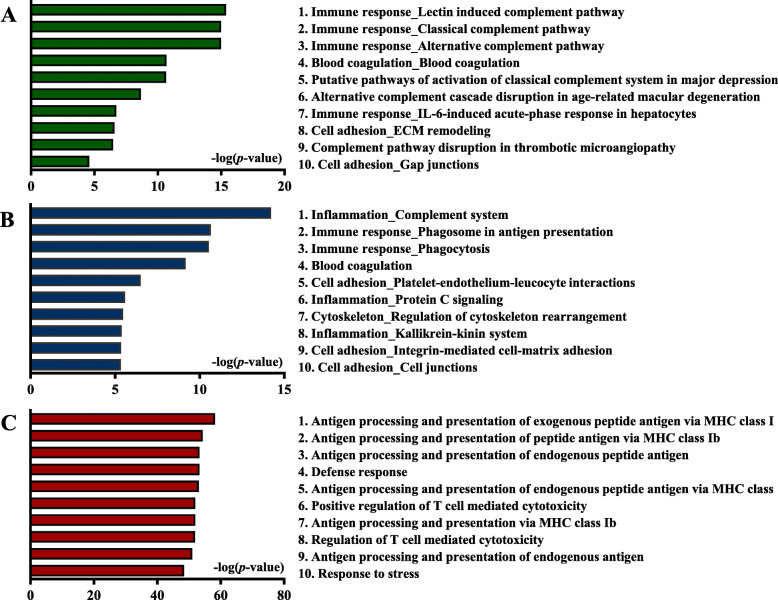
Process network analysis for differentially expressed proteins between healthy controls and rheumatoid arthritis (RA) patients. **a** Pathway map, process networks, and Gene Ontology processes associated with differentially expressed proteins between healthy controls and RA patients. **b** The most significant process networks between healthy controls and RA patients. The process network with the lowest *p* value was the complement system. **c** The most significant pathway map between healthy controls and RA patients. The pathway map with the lowest *p* value was the lectin-induced complement pathway

First, as indicated in the volcano plot, we selected significantly upregulated or downregulated proteins by > 2-fold (*p* < 0.05) (Fig. 1b). Heatmap analysis revealed differentially expressed proteins in the two groups (Fig. 1c). Furthermore, we performed Gene Ontology (GO) analysis of proteins with FC > 2.0 and *p* < 0.05 between healthy controls and RA patients. The three most significantly enriched pathways were complement-related pathways associated with immune responses, including the lectin-induced complement pathway, the classical complement pathway, and the alternative complement pathway (Fig. 2a). Moreover, three pathways were significantly associated with immunity and inflammation, including the complement system, phagosome involvement in antigen presentation, and phagocytosis (Fig. 2b). On GO analysis of biological processes, the three most significant pathways were antigen processing and presentation of exogenous peptide antigens via MHC class I, antigen processing and presentation of peptide antigens via MHC class Ib, and antigen processing and presentation of endogenous peptide antigens (Fig. 2c).

**Fig. 3:**
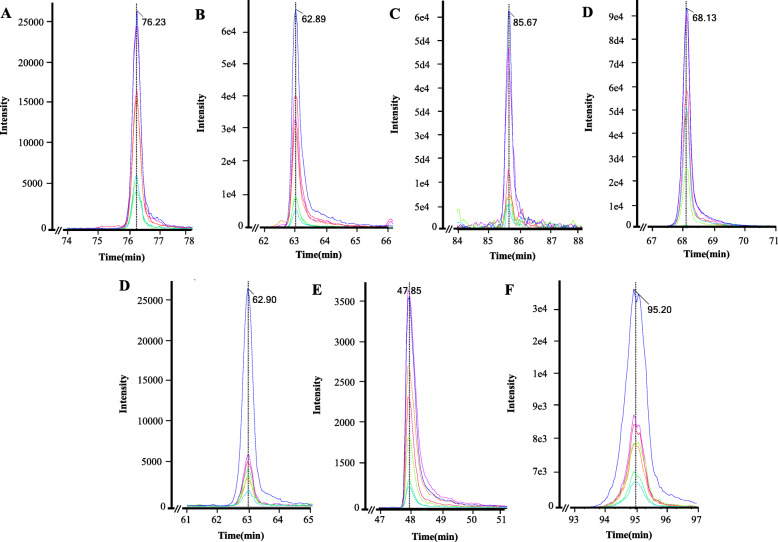
Chromatography of selected candidate proteins extracted from RA patients. **a**–**g** Extract ion chromatography of the peptides from seven candidate proteins including angiotensinogen, C-reactive protein, gelsolin, lymphatic vessel endothelial hyaluronan receptor 1, retinol-binding protein 4, serum amyloid A-4, and vitamin D-binding protein

On protein quantification of individual serum samples, we selected seven candidate biomarkers, which were then subjected to MRM absolute quantification, including angiotensinogen, C-reactive protein, gelsolin, lymphatic vessel endothelial hyaluronan receptor 1, retinol-binding protein 4, serum amyloid A-4, and vitamin D-binding protein (VDBP) (Fig. 3). For MRM analysis of the seven selected candidate biomarkers, one peptide was selected per protein, and after optimization, the parameters were selected.

**Fig. 4:**
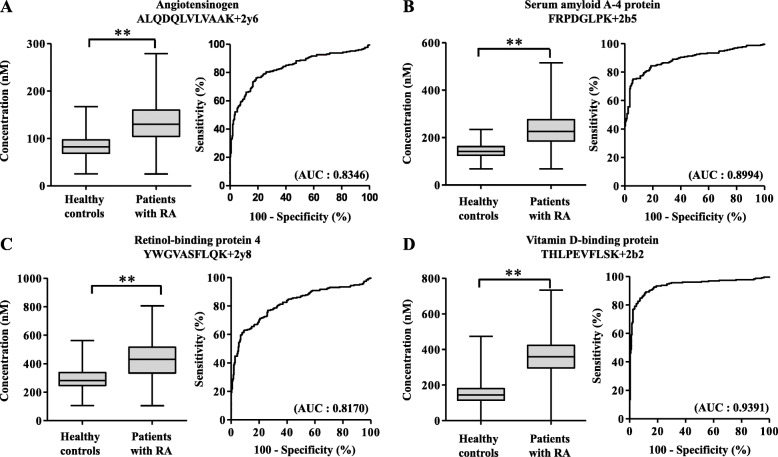
Box and whisker plots of selected biomarker candidates in healthy controls and rheumatoid arthritis (RA) patients. Proteins altered among RA patients relative to the healthy controls were selected. **a**–**d** Angiotensinogen, serum amyloid A-4 protein (SAA4), retinol-binding protein-4 (RBP4), and vitamin D-binding protein (VDBP) profiles were compared between healthy controls and RA patients. The number of healthy controls and RA patients was 251 and 230, respectively. Box plots represent the upper quartile, lower quartile, and median (horizontal line). Whiskers enclose the range (min-max value). Independent samples *t* tests were used to determine the statistical significance **p < 0.001. AUC, area under the curve

Of the seven proteins identified, those with an area under the curve (AUC) > 0.8 were angiotensinogen (AUC = 0.8346), serum amyloid A-4 (AUC = 0.8994), VDBP (AUC = 0.8170), and retinol-binding protein 4 (AUC = 0.9391) (Fig. 4), whereas lymphatic vessel endothelial hyaluronan receptor 1, gelsolin, and C-reactive protein revealed AUC values of 0.5309, 0.6794, and 0.5030, respectively.

**Fig. 5:**
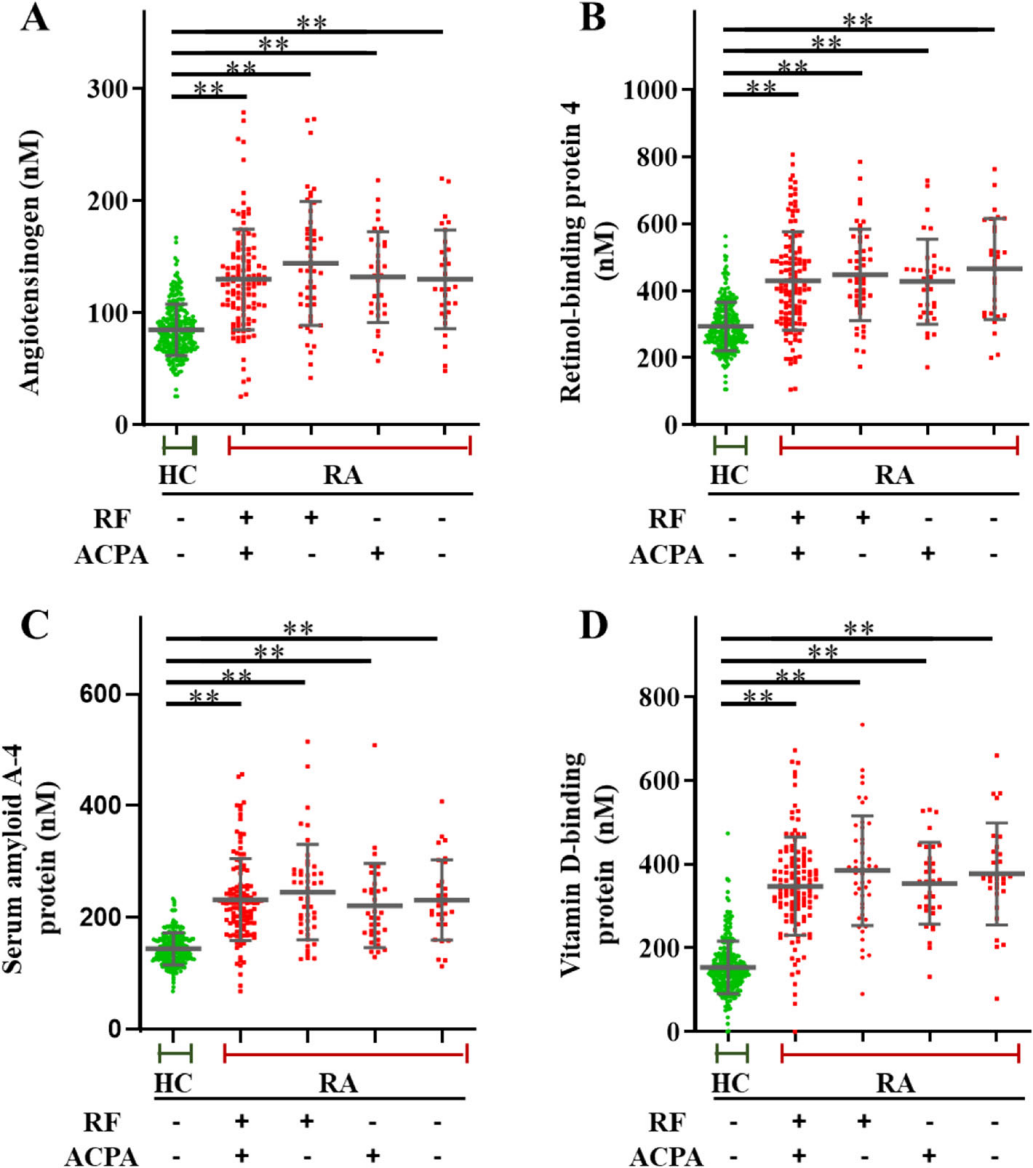
Scatter plots of selected biomarker candidates in healthy controls and RF- and anti-CCP-positive/negative RA. Proteins altered among rheumatoid (RF)- and anti-CCP (ACCP)-positive or -negative RA patients relative to those in the healthy controls were selected. **a**–**d** Angiotensinogen, retinol-binding protein-4 (RBP4), serum amyloid A-4 protein (SAA4), and vitamin D-binding protein (VDBP) profiles were compared between healthy controls and RA patients in accordance with the antibody titer. The number of healthy controls and RA patients was 251 and 230 (RF+/ACPA+ *n* = 121, RF+/ACPA− *n* = 47, RF−/ACPA+ *n* = 33, RF−/ACPA− *n* = 29), respectively. Plots indicate individual protein abundance in each group. Data are presented as mean ± SD values. Independent samples *t* tests were used to determine the statistical significance **p < 0.001. HC, healthy controls; RA, patients with rheumatoid arthritis

**Fig. 6:**
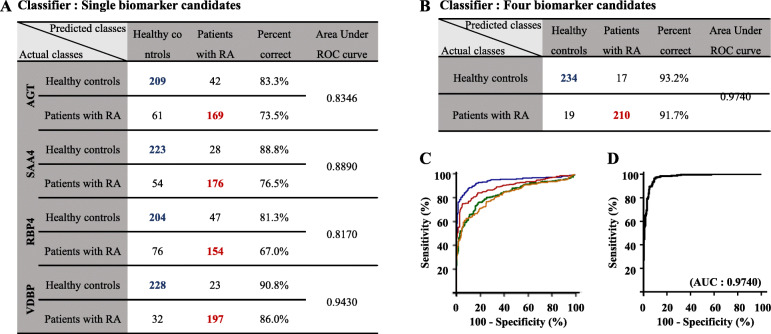
Logistic regression analysis for selected biomarker candidates in healthy controls and rheumatoid arthritis (RA) patients. Predictive accuracy of single (**a**) and the four biomarker candidates (**b**). Receiver operating characteristic curve (ROC) analysis of single (**c**) and four biomarker candidates (**d**) was performed. The number of healthy controls and RA patients was 251 and 230, respectively. The plots indicate individual protein abundance in each group. Data are presented as mean ± SEM values. Independent samples *t* tests were used to determine the statistical significance

Furthermore, RA patients were categorized as RF-positive, RF-negative, ACCP-positive, and ACCP-negative. These four biomarker candidates displayed high classification accuracy regardless of the RF-positive or RF-negative status of patients (Fig. 5). Furthermore, logistic regression analysis was performed to predict the classification accuracy among healthy controls and RA patients. Consequently, angiotensinogen (AGT) accurately classified 209 healthy controls and 169 RA patients in predicted classes from among 250 healthy controls and 230 RA patients in actual classes. The classification accuracy for healthy controls and RA patients was 83.3% and 73.5%, respectively (Fig. 6a). Furthermore, serum amyloid A-4 (SAA4) accurately classified 223 healthy controls and 176 RA patients in predicted classes, with a classification accuracy of 88.8% and 76.5% for healthy controls and RA patients, respectively (Fig. 6a). Retinol-binding protein-4 (RBP4) accurately classified 204 healthy controls and 228 RA patients in predicted classes, with a classification accuracy of 90.8% and 86.0% for healthy controls and RA patients, respectively (Fig. 6a). Vitamin D-binding protein (VDBP) accurately classified 228 healthy controls and 197 RA patients in predicted classes, with a classification accuracy of 90.8% and 86.0% for healthy controls and RA patients, respectively (Fig. 6a). Together, the four-biomarker panel accurately classified 234 healthy controls and 210 RA patients with a classification accuracy of 93.2% and 91.7% for healthy controls and RA patients, respectively (Fig. 6b). The AUC values of the four individual biomarkers were 0.8346, 0.8890, 0.8170, and 0.9430 (Fig. 6c), and of the four-biomarker panel, 0.9740 (Fig. 6d).

## Discussion

In this study, we analyzed the serum proteins in 251 healthy controls and 230 patients with RA to identify diagnostic biomarkers through MS analysis. Analysis of individual serum samples revealed differentially expressed proteins in the two groups, among which, AGT, RBP4, SAA4, and VDBP emerged as novel diagnostic biomarkers on MRM absolute quantification, and their AUC value was over 0.8, indicating a high diagnostic efficiency.

We analyzed proteins significantly upregulated by > 2-fold (*p* < 0.05). A pathway map of the functional analysis revealed that the three main complement pathways were associated with these differentially expressed proteins. Moreover, the complement system was the most significantly associated pathway with RA upon GO analysis of biological processes. In the lectin-induced complement pathway, complement proteins including C3, C3a, C3b, C4, C4a, C4b, C5, C5a, C5b, complement C3d receptor 2 (CD21), ficolin 3, and iC3b were downregulated by > 8-fold among RA patients. Likewise, expression patterns of the proteins involved in the classical and lectin-induced complement pathways were similar in patients with RA. However, the C1s complement protein was upregulated > 9-fold in RA patients in the classical pathway and vitronectin and by > 8-fold in the alternative pathway. Complement C1s protein is an early component of the classical pathway and initiates the complement pathway and is reportedly associated with the degeneration of articular cartilage in RA. Meanwhile, inhibitory protein alpha 1-antitrypsin of the complement pathway inducing inflammation was upregulated in patients with RA [19]. Furthermore, alpha 1-antitrypsin inhibits thrombin activity and blood coagulation as well as inhibition of inflammation by complement pathway control, suggesting that aberrant blood coagulation initiated in RA can be attenuated through alpha 1-antitrypsin overexpression.

VDBP is mainly produced in the liver. When tissue damage occurred, increased permeability of cells releases polymerization of F-actin, leading to a blocked blood vessel in RA. Along with tissue damage, VDBP was immediately released from the damaged cell [20]. Increased serum VDBP is suggested to play a role in scavenging actin and inhibiting the negative effects of F-actin. Besides, the VDBP-G-actin complex was involved in neutrophil migration, suggesting VDBP overexpression might allow proteins to act immediately and directly during RA-induced tissue damage [20]. However, vitamin D activated by VDBP protects against joint tissue damage during RA, owing to its anti-inflammatory effects [20]. 25-Hydroxyl vitamin D is activated by VDBP and moves to immune cells of several organs, inducing an anti-inflammatory effect [21]. RA is three times more prevalent in women than in men. The well-established evidence on the prevalence in women is the association between the female sex hormone such as estrogen and RA. The increase in estrogen has been reported to alleviate the onset of RA. Interestingly, VDBP is upregulated by the increase in estrogen, thus playing an important role in the anti-inflammatory activity and tissue recovery [12, 22]. Likewise, the role of VDBP in the pathogenesis of RA can be interpreted from both anti- and pro-inflammation.

SAA4 is an acute-phase protein reportedly upregulated in RA [23]. SAA4 is activated by cytokines such as IL-1, IL-6, and TNF-alpha and has pro-inflammatory effects. Moreover, SAA4 positively correlated with C-reactive protein [24]. Furthermore, SAA4 had a better efficacy for diagnosis than C-reactive protein being used for the diagnosis of RA [24]. In this study, SAA4 was also identified as a candidate biomarker for the diagnosis of RA, and the results of MRM absolute quantification were concurrent with those from previous studies.

The renin-angiotensin system is associated with the inflammatory response and helps maintain blood pressure [25]. Angiotensin II mediates inflammation by stimulating immune cells [26]. For example, angiotensin II regulates pro-inflammatory transcription factor nuclear factor-κB [27]. AGT is an angiotensin II precursor [28]. This study revealed the effects of the renin-angiotensin system on inflammatory reactions. An increase in angiotensinogen in serum samples of RA patients is associated with the renin-angiotensin system comprising AGT and angiotensin II [15].

RBP4s, called retinol-binding protein (RBP), are transport proteins for retinol (vitamin A_1_). Retinol is synthesized in the liver and circulated into the blood by RBP [29]. RBP is associated with insulin resistance, obesity, and cardiovascular disease [30, 31]. Previous studies have reported that RBP4 is upregulated in insulin-resistant mice and is upregulated in the serum of patients with obesity or type 2 diabetes, thus affecting insulin signaling [32–34]. Likewise, RBP4 was reported as the predictor of atherosclerosis in patients with RA [35]. However, the association between elevated RBP and RA pathology has not been defined. Although, in a previous study on RA biomarkers, it has been reported that RBP4 is a candidate RA biomarker through ELISA [22].

In correlation analysis between the four biomarkers and conventional blood biomarker, there is no correlation between either the candidate proteins or autoantibody/inflammation markers. As mentioned above, candidate biomarkers are likely to be related with inflammation and dysfunctional immune systems. However, in our study, there is no mechanistic evidence to prove the association between the biomarkers and RA pathology. Thus, further investigation is needed to clarify the mechanism related with the biomarkers for clinical application.

The candidate proteins have a distinction between comparative groups; however, among the four candidate biomarkers, VDBP have a distinction in individual patients and the difference in the mean between the comparison groups. The range of the mean ± SD of the patient and control groups does not overlap. However, individual patients who are not present within ± SD may be confused with the normal group, leading to a false negative. Therefore, to better differentiate RA patients from healthy controls, in further studies, it is necessary to analyze the classified patient group from various clinical perspectives. For example, disease stage reflecting inflammation response, smoking, and estrogen concentration may affect the expression of VDBP [36]. This classified sample analysis is expected to enable personalized diagnosis and optimal treatment as well as improve diagnostic efficacy.

## Conclusions

This study shows that four proteins validated through MRM were analyzed among RF-positive, RF-negative, ACCP-positive, and ACCP-negative RA patients to confirm their potential to distinguish RA patients from healthy controls regardless of the titer of RF and ACCP. RF is an existing RA diagnostic marker; however, it has limitations associated with RA diagnosis, including a low sensitivity of 60% and a specificity of 85%. Furthermore, RF has been detected in non-RA diseases, thus deterring an accurate diagnosis of RA. Therefore, to increase the diagnostic efficiency of RA, anti-CCP is used; however, anti-CCP has a similar or higher specificity and sensitivity than RF. Hence, we identified four candidate biomarkers including angiotensinogen, SAA4, RBP4, and VDBP, which could significantly distinguish RF-positive, RF-negative, ACCP-positive, and ACCP-negative RA patients, and particularly the seronegative (RF- and ACCP-negative) patients. Therefore, a combination of these four markers can diagnose RA with greater accuracy, serving as highly robust biomarkers along with RF and ACCP.

## Data Availability

The datasets used and/or analyzed during the current study are available from the corresponding author upon reasonable request.

## Abbreviations

RA: Rheumatoid arthritis
RF: Rheumatoid factor
anti-CPP: Anti-cyclic citrullinated peptide
ACCP: Anti-CCP
CRP: C-reactive protein
MRM: Multiple-reaction-monitoring-based
RBP-4: Retinol-binding protein-4
SAA4: Serum amyloid A-4 protein
VDBP: Vitamin D-binding protein
LYVE1: Lymphatic vessel endothelial hyaluronan receptor 1
CE: Collision energy
DP: Declustering potential
CXP: Cell exit potential
PCA: Principal component analysis
GO: Gene Ontology
CD21: Complement C3d receptor 2
AUC: Area under the curve
